# Carbocyclic Analogues of Inosine-5’-Monophosphate: Synthesis and Biological Activity 

**Published:** 2012

**Authors:** E.S. Matyugina, S.N. Andreevskaya, T.G. Smirnova, A.L. Khandazhinskaya

**Affiliations:** Engelhardt Institute of Molecular Biology, Russian Academy of Sciences, Vavilova Str., 32, Moscow, Russia, 119991; Central Tuberculosis Research Institute, Russian Academy of Medical Sciences, 2, Yauzskaya Alley, Moscow, Russia, 107564

**Keywords:** **Carbocyclic nucleosides, competitive inhibition, inosine-5’-monophosphate, human IMPDH II,*Mycobacterium tuberculosis*

## Abstract

9-(4’-Phosphonomethoxy-2’-cyclopenten-1’-yl)hypoxanthine and 9-(4’-phosphonomethoxy-2’,3’-dihydroxycyclopenten-1’-yl)hypoxanthine were synthesized as isosteric carbocyclic analogues of inosine-5’-monophosphate. The synthesized compounds were shown to be capable of inhibiting the activity of human type II inosine-5′-monophosphate dehydrogenase (IMPDH II) (IC_50 _= 500 µM) and to have no significant effects on the growth of*Mycobacterium tuberculosis*.

## INRODUCTION 

Inosine monophosphate dehydrogenase (IMPDH, [EC 1.1.1.205]) is one of the key enzymes in a *de novo* biosynthesis of purine nucleotides (GTP and dGTP). Inosine-5’-monophosphate (IMP) is the natural substrate of IMPDH. IMPDH catalyzes NAD ^+^ -dependent reactions leading to the formation of NADH and xanthosine-5’-monophosphate, which is then converted into guanosine-5’-monophosphate (GMP). The inhibition of IMPDH causes a decrease in the intracellular levels of guanine-containing nucleotides, leading to antimicrobial, antiparasitic, antiviral, anticancer, and immunodepressive effects [1, 2]. 

The existing inhibitors of IMPDH can be classified into 3 groups with respect to enzyme binding: analogues of IMP, analogues of NAD ^+^ ,and allosteric inhibitors. The modified nucleosides belonging to the first group undergo intracellular phosphorylation and, in the form of 5’-monophosphates, competitively interact with the IMP binding site. Both types of inhibitors exist among the analogues of IMP: reversible (ribavirin-5’-monophosphate, 3’-deazaguanosine, mizoribin) and irreversible (5’-monophosphates of 2-vinyl inosine, 6-chloropurine nucleoside, 5-ethinyl-1-ribofuranosyl-imidazole-4-carboxamide). The most widely known representatives of the second group of inhibitors include tiazofurine, selenazofurine, and mycophenolic acid. 

Human IMPDH exists in 2 isoforms, types I and II, showing 84% homology. Type I enzyme is prevalent in normal lymphocytes and leucocytes; type II is found mostly in actively dividing and cancerous cells. Bacterial IMPDH molecules isolated from different sources significantly differ from the human enzyme, showing 30-41% homology. The affinity of IMPDH isolated from different sources may vary significantly for the same types of inhibitors [2]. Thus, human IMPDH type II is more sensitive to mycophenolic acid (K _i_ = 7nM) as compared to human IMPDH type I (K _i_ = 33nM). The sensitivity of bacterial enzymes to mycophenolic acid is considerably lower ( *K*_i_ = 0.2–20 µM). The selectivity of IMPDH with respect to inhibitors makes this enzyme a rather attractive target for potential anticancer, antimicrobial, and antiparasitic compounds [2]. 

It has been shown recently that the inhibition of IMPDH isolated from *Mycobacterium tuberculosis* suppresses the growth of the bacterium [3]. The main objective in efforts to treat tuberculosis today is searching for new drugs that are effective against strains resistant to existing medicinal agents. New compounds should work via different mechanisms compared to those of the existing therapeutic agents. Hence, searching for new anti-tuberculosis agents not only among classes of antibiotics, but also among compounds of another nature seems reasonable. There are no analogues of nucleosides among the therapeutic agents currently used to treat tuberculosis; in combination with their potential to inhibit IMPDH, it makes these analogues primary candidates for investigation as antimycobacterial agents. 

**Fig. 1:**
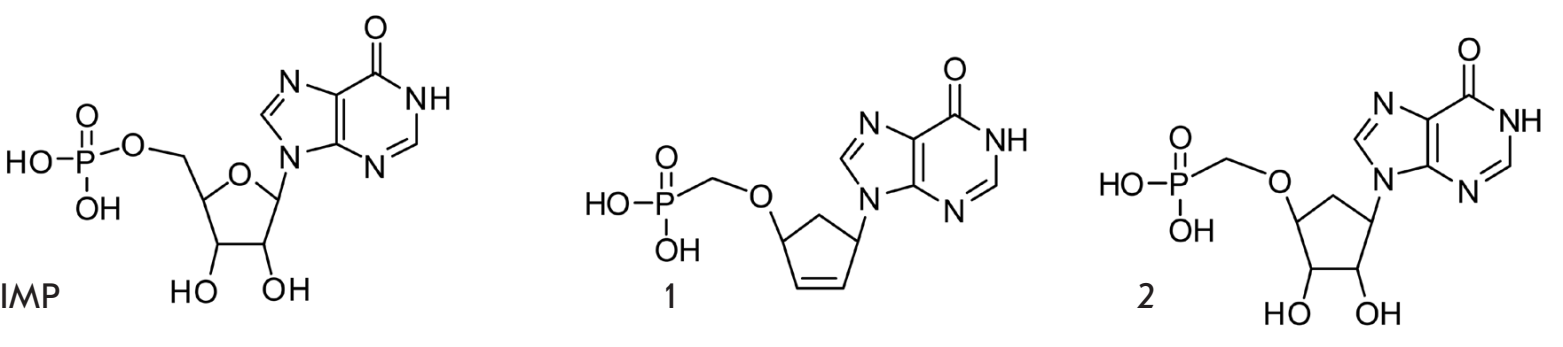
Inosine-5’-monophosphate and its isosteric carbocyclic analogues

The present article describes the synthesis of 9-(4’-phosphonomethoxy-2’-cyclopenten-1’-yl)hypoxanthine (1) and 9-(4’-phosphonomethoxy-2’,3’-dihydroxycyclopenten-1’-yl)hypoxanthine (2) (Fig. 1), the isosteric carbocyclic analogues of IMP. The ability of these compounds to inhibit human IMPDH II and to suppress the growth of *M. tuberculosis * is also assessed. 

## EXPERIMENTAL 

All the reagents and solvents used in the experiments are commercially available (Acros, Aldrich, and Fluka). Thin-layer chromatography (TLC) was performed on Kieselgel 60 F _254 _ plates (Merck) in the following systems: 98:2 CHCl _3_ –MeOH (system А); 9:1 CHCl _3_ –MeOH (system B), 4:1 dioxane–NH _3_ (system C), 7:2:1 isopropanol–NH _3_ –water (system D). Column chromatography was performed using a 40-63 µm Kieselgel (,Merck), a 25-40 µm Lichroprep RP-18 (Merck), and a DEAE-Toyopearl (Toysoda, Japan). The elution systems are specified below. 

The UV spectra were recorded using a Shimadzu UV-1201 spectrophotometer (Japan). ^1^ H and ^31^ P NMR spectra were recorded on an AMX III-400 spectrometer (Bruker) with operating frequencies of 400 MHz for ^1^ Н NMR (chemical shifts relative to the internal standards are provided: Me _4_ Si for organic solvents and sodium 3-(trimethylsilyl)-1-propansulfonate (DSS) for D _2_ O) and 162 MHz for ^31^ Р NMR (with suppression of phosphorus-proton spin-spin coupling; chemical shifts relative to the external standards, 85% phosphoric acid, are provided). Chemical shifts are given in parts per million (ppm). 

The starting 6-chloro-9-(4’-hydroxy-2’-cyclopenten-1’-yl)purine (3) was synthesized in accordance with the previously described methodology [4]. 

**6-Ethoxy-9-(4’-hydroxy-2’-cyclopenten-1’-yl)purine (4).**

CalcinedК _2_ СО _3 _ (300 mg, 2.3 mmol) was added to a solution of9-(4’-hydroxy-2’-cyclopent-1’-yl)-6-chloropurine (300 mg, 13 mmol) dissolved in 10 ml of ethanol; the resultant suspension was refluxed for 1 h. The course of the reaction was controlled using TLC (system A). 

The solvent was removed under reduced pressure; the residue was applied onto a silica gel column; system B was used for elution; the target fractions were concentrated under reduced pressure. Product **4** (220 mg, 78%) was isolated as a white foamy substance. ^1^ H NMR (CD _3_ OD): 8.42 (1H, s, H _2_ ), 7.95 (1H, s, H _8_ ), 6.34–6.33 (1H, m, H _2’_ ), 5.82 (1H, m, H _3’_ ), 5.34–5.32 (1H, m, H _1’_ ), 4.86 (1H, m, H _4’_ ), 4.64–4.62 (2Н, m, О-СН _2_ ), 3.02–2.98 (1H, m, H _а5_ ), 2.23–2.19 (1H, m, H _b5_ ), 1.5–1.48 (3Н, m, СН _3_ ). 

**6-Ethoxy-9-(4’-ethylphosphonomethoxy-2’-cyclopenten-1’-yl)purine (5). **

NaH (33.5 mg, 1.4 mmol) and Cs _2_ CO _3 _ (234 mg, 0.72 mmol) were added to the solution of compound **4** (230 mg, 0.93 mmol) in 5 ml of dimethylformamide (DMF) under stirring in an argon atmosphere. The reaction mixture was stirred for 1.5 h at room temperature, then ethyl ester of *p* -toluene sulfonyloxymethyl phosphonic acid (334 mg, 1.8 mmol) solution in DMF (2 ml) was added. The mixture was stirred for 12 h at room temperature. The course of the reaction was controlled using TLC (system B). After removing the solvent under reduced pressure, the residue was applied onto the DEAE-Toyopearl column and eluted with a linear gradient of NH _4_ HCO _3_ (0–0.2 M). The target compound **5** was eluted using 0.1 M NH _4_ HCO _3_ ; the fraction was concentrated, and the target product was isolated on a LiChroprep RP-18 and eluted using a linear gradient of aqueous ethanol (0–10%). The product was eluted using an 8% aqueous ethanol solution.

A total of 240 mg (67%) of compound **5 ** was obtained in the form of a colorless oil. ^1^ Н NMR (D _2_ O): 8.14 (1H, s, H _2_ ), 8.06 (1H, s, H _8_ ), 6.34–6.32 (1H, m, H _2_ ), 6.15 (1H, m, H _3_ ), 5.35 (1H, m, H _1_ ), 4.63 (1H, m, H _4_ ), 4.38 (2Н, m, О–СН _2_ ), 3.76–3.72 (2Н, m, О–СН _2_ ), 3.58–3.56 (2Н, m, О–СН _2_ –Р), 2.89 (1H, m, H _а5_ ), 1.80 (1H, m, H _b5_ ), 1.33–1.29 (3Н, m, СН _3_ ), 1.15–1.11 (3Н, m, СН _3_ ). ^31^ Р NMR (D _2_ O): 17.99 s. 

**6-Ethoxy-9-(4’-ethylphosphonomethoxy-2’,3’-dihydroxycyclopent-1’-yl)purine (6). **

Solutions of osmium tetroxide in dioxane (0.5 M) and N-methyl morpholine oxide (0.3 ml, 3 mmol) were added to the suspension of phosphonate **5** (200 mg, 0.54 mmol) in a 10:1 mixture of dioxane and water solvents. The solution was stirred for 3 h at room temperature. The course of the reaction was controlled using TLC (system D). After the solvent had been removed under reduced pressure, the residue was applied onto the DEAE-Toyopearl column and eluted with a linear gradient of NH _4_ HCO _3_ (0–0.3 M), and subsequently repurified on a Lichroprep RP-18 column eluted with water. The yield of product **6** was 74%. The UV spectra (Н _2_ О,рН 7) λ _max_ 252.0 nm (ε 9600), ^1^ Н NMR (D _2_ O): 8.36 (1H, s, H _2_ ), 8.29 (1H, s, H _8_ ), 4.85 (1H, m, H _1_ ), 4.23 (1H, m, H _4_ ), 3.92–3.89 (2Н, m, О–СН _2_ ), 3.73 (2Н, m, О–СН _2_ ), 3.60–3.59 (2Н, m, О–СН _2_ –Р), 2.88–2.85 (1H, m, H _а5_ ), 2.10 (1H, m, H _b5_ ), 1.37 (3Н, m, СН _3_ ), 1.21 (3Н, m, СН _3_ ). ^31^ Р NMR (D _2_ O): 18.23 s. 

**9-(4’-Phosphonomethoxy-2’-cyclopenten-1’-yl)hypoxanthine (1).**

**Scheme:**
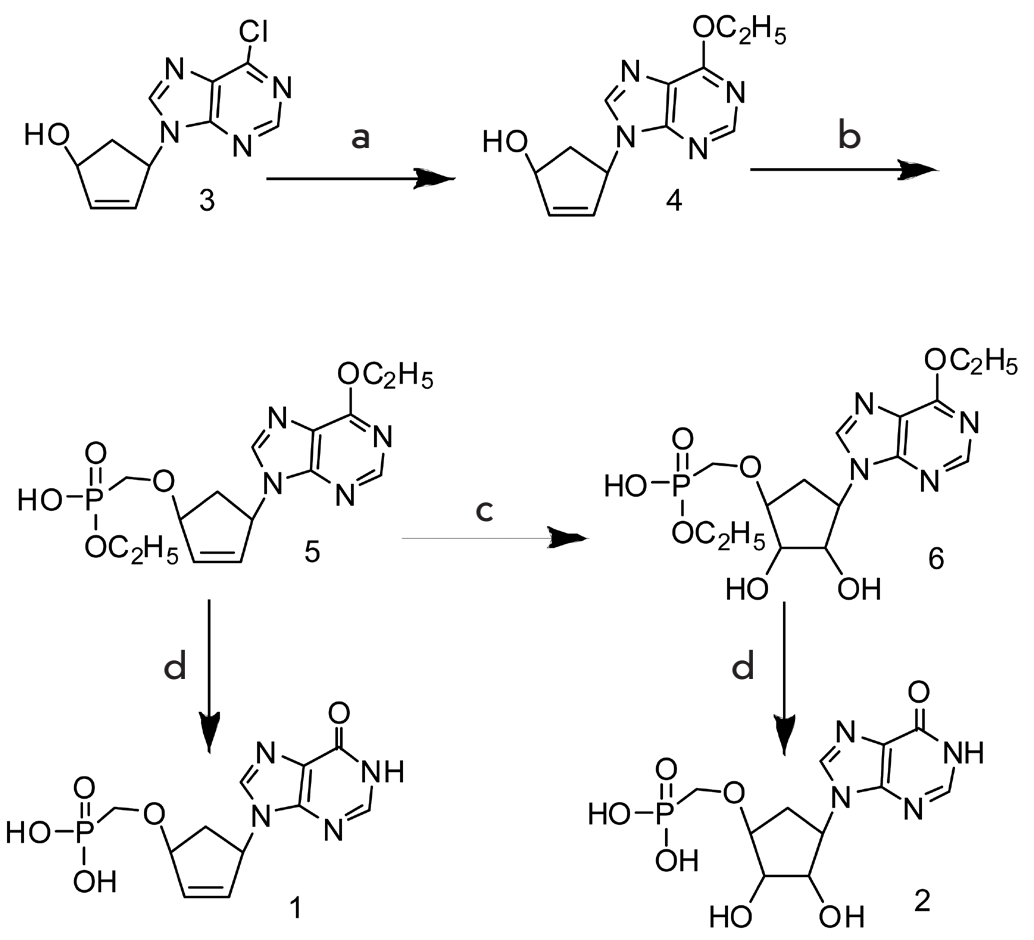
a – Ethanol (pure), K _2_ CO _3_ , refluxing for 1 hour; b – NaH, TosOCH _2_ P(O)OC _2_ H _5_ OH, Cs _2_ CO _3_ , DMF; c – OsO _4_ , NMMO, dioxane; d - (CH _3_ ) _3_ SiBr, DMF

Trimethylbromosilane (0.65 ml, 5 mmol) was added to the suspension of phosphonate **5** (100 mg, 0.27 mmol) in DMF under argon atmosphere; the resulting mixture was stirred for 3 h at room temperature. The course of the reaction was controlled using TLC (system B). The reaction mixture was neutralized with 25% aqueous ammonia; the solvent was removed under reduced pressure. The residue was purified on a Lichroprep RP-18 column and eluted with water to give 70 mg (84%) of product **4** in the form of lyophilizate. ^1^ Н NMR (D _2_ О): 8.39 (1H, s, H _2_ ), 8.26 (1H, s, H _8_ ), 6.44–6.42 (1H, m, H _2’_ ), 6.18–6.17 (1H, m, H _3_ ), 5.57–5.55 (1H, m, H _1_ ), 4.81 (1H, m, H _4_ ), 3.61 (2Н, m, О–СН _2_ –Р), 3.04 (1H, m, H _а5_ ), 1.93 (1H, m, H _b5_ ). ^31^ Р NMR (D _2_ O): 16.66 s. 

**9-(4’-phosphonomethoxy-2’,3’-dihydroxycyclopent-1’-yl)hypoxanthine (2). **

The compound **2** was obtained in a similar fashion to compound **1,** obtained from compound **6** (140 mg, 0.35 mmol). A total of 105 mg (81%) of the product was isolated as lyophilizate. The UV spectra (Н _2_ О,рН 7) λ _max_ 251.0 nm (ε 9300). ^ 1^ Н NMR (D _2_ О): 8.27 (1H, s, H _2_ ), 8.11 (1H, s, H _8_ ), 4.20 (1H, m, H _1_ ), 3.93 (1H, m, H _4_ ), 3.53–3.51 (2Н, m, О–СН _2_ –Р), 2.81 (1H, m, H _а5_ ), 2.07 (1H, m, H _b5_ ). ^31^ Р NMR (D _2_ O): 14.06 s. 

## BIOLOGICAL TESTS 

Experiments on the ability of the synthesized compounds to inhibit human IMPDH II were conducted by NovoCib company (France). Compounds **1** and **2** were tested on a human recombinant IMPDH II (~0.0003 activity units per well) at 37°C in 200 µl of a buffer solution (KH _2_ PO _4_ 0.1 M, pH 7.8, NAD 250 µM, DTT 2 mM) using a 96-well microplate. The reaction was initiated by the addition of a substrate, IMP, at a concentration of 250 µM. Prior to reaction initiation, the compounds were incubated in a buffer with IMPDH II for 5 minutes. The absorbance was measured at 340 nm using an iEMS Reader MF device (Labsystems, Finland). Ribavirin was used as a positive control. The influence of the synthesized compounds on human IMPDH II activity was simultaneously tested in two identical experiments. 

**Antituberculosis activity.**

The compounds were tested on a laboratory strain of *M. tuberculosis* H37Rv sensitive to antituberculosis drugs. The mycobacteria was transferred into a single-cell suspension of single cells at the same growth phase and standardized with respect to CFU [5]. The enriched liquid growth medium Dubois (Difco, USA) was used. 

**Evaluation of compound efficacy. **

The effect of the compounds on the growth of mycobacterial strains was estimated using a Bactec MGIT 960 automated growth detection system (BD, USA). The suspension of mycobacterial cells (500 µl) was inoculated in a nutrient medium (7.9 ml). The final concentration of *M. tuberculosis* in the sample was 10 ^6^ CFU/ml. Three replicates for each sample (concentration) were analyzed, including the control samples. The antimicrobial activity of the compounds was evaluated using the proportion method with the TB Exist software [6]. The growth of mycobacterial cells cultured in the presence of the compound and the growth of the control culture diluted 100 times as compared to the test sample is assessed in this analysis. The culture is considered to be sensitive to such a concentration of the compound at which the growth rates in the experiment do not exceed 100 growth units (GU), when 400 GU are recorded in the control sample; the compound is regarded as active in this case. Furthermore, the absolute concentration method was used to assess the effect of the compounds at concentrations lower than the minimum inhibitory concentration (MIC) on the viability of mycobacterial cells on the basis of the inhibition of bacterial growth as compared to the control. Bacterial growth was determined automatically at 1 h intervals and recorded using the Epicenter software (BD, USA). 

## RESULTS AND DISCUSSION 

**Fig. 2:**
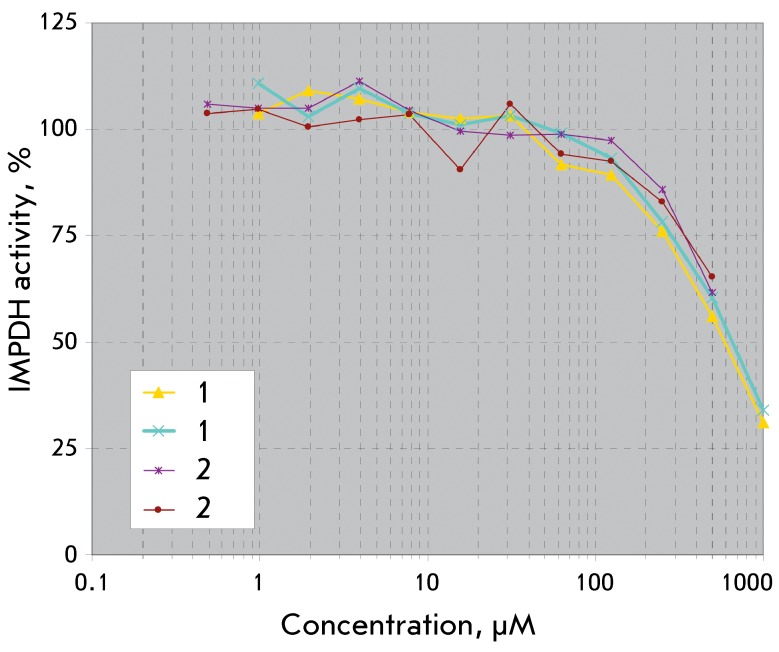
Dose-dependent inhibition of IMPDH II by compounds 1 and 2

Over the past decades, carbocyclic nucleosides have been intensively studied. These compounds have been found to be biologically active; in particular, they turn out to have antiviral and anticancer properties [7]. These nucleosides are recognized by many enzymes and receptors, since their structure is similar to that of natural nucleosides. Meanwhile, they are highly resistant to C-N bond cleavage by phosphorylases and hydrolases. 

Hydroxycyclopentene is used as a carbocyclic moiety in compounds **1** and **2** . The analogues with such moiety are known as 5’-norcarbocyclic nucleosides. The substitution of the primary 5’-hydroxyl residue for the secondary 4’-hydroxyl results in toxicity decrease due to the loss of substrate properties with respect to cellular kinases. Taking into account the fact that intracellular phosphorylation of 5’-norcarbocyclic nucleosides is infeasible, we synthesized the methylene phosphonates of 9-(4’-hydroxy-2’-cyclopenten-1’-yl)hypoxanthine and 9-(4’,2’,3’-trihydroxycyclopent-1’-yl)hypoxanthine ( *scheme* ). It was previously shown that such isosteric phosphonates imitate the corresponding monophosphates but are more stable to the action of hydrolyzing and dephosphorylizing enzymes [8]. 

9-(4’-Hydroxy-2’-cyclopenten-1’-yl)-6-chloropurine **3** was obtained via condensation of epoxycyclopentene and 6-chloropurine in accordance with the previously described procedure [4].Refluxing of compound **3 ** in ethanol led to the formation of ester **4** , which subsequently reacted with the ethyl esters of tosyloxymethylphosphonic or iodomethylphosphonic acid, to yield monophosphonate **5** . ****

Ester **5** was hydrolyzed to 9-(4’-phosphonylmethyloxy-2’-cyclopenten-1’-yl)hypoxanthine **1** using excess amounts of trimethylbromosilane. In order to obtain monophosphonate **2,** the double bond of compound ** 5 ** was oxidized with osmium tetroxide in the presence of N-methylmorpholine-N-oxide, and the ethyl groups were subsequently removed from compound **6** in the presence of trimethylbromosilane ( *scheme* ). The target compounds **1** and **2 ** were purified on a DEAE-Toyopearl column eluted with a linear concentration gradient of NH _4_ HCO _3_ . The subsequent purification and removal of salts was performed on a Lichroprep RP-18 column. The final yields were 84 and 81%, respectively. 

Compounds **1** and **2 ** were tested as human IMPDH II inhibitors (Fig. 2). 

It is clear from *Fig. 2* that the carbocylic analogue **1 ** at a concentration of 500 µM inhibited enzymatic activity by 50% ( *K*_i _ 474 µМ), whereas compound **2** did so by 35–39% ( *K*_i _ 975 µМ). Ribavirin monophosphate was used as the control and at a concentration of 2 µМ inhibited the enzymatic activity by 50%; the *K*_m _ value of the IMP (natural substrate) in this system was 124.4 µМ. 

The ability of the monophosphonates **1** and **2** to inhibit the growth of a *M. tuberculosis * was also tested. The growth of *M. tuberculosis* culture H37Rv under the action of compounds **1** and **2** at concentrations of 2–100 µg/ml (5–320 µМ) was identical to that observed in the control group: the initial phases of culture growth were detected on day 7; entry into stationary phase was detected on day 17. The duration of active bacterial growth is 10 days. Compound **2,** at concentrations of 200 µg/ml (578 µМ), caused an insignificant delay (2 days) in bacterial growth as compared to the control group. 

## CONCLUSIONS 

Synthesized 9-(4’-phosphonomethoxy-2’-cyclopenten-1’-yl)hypoxanthine and 9-(4’-phosphonomethoxy-2’,3’-dihydroxycyclopent-1’-yl)hypoxanthine are weak inhibitors of human IMPDH II. These compounds at concentrations of 20–200 µg/ml do not affect the growth of *M. tuberculosis* H37Rv *in vitro* . This fact can be attributed both to the structural features of the mycobacterial cell wall and, hence, the difficulties associated with penetrating the membrane, or to the existence of alternative pathways for synthesizing essential compounds in mycobacteria. The hypothesis that IMPDH of *M. tuberculosis* could be less sensitive to the compounds under study compared with human IMPDH II should not be dismissed, either. 
